# Green tea polyphenol epigallocatechin-*O*-gallate induces cell death by acid sphingomyelinase activation in chronic myeloid leukemia cells

**DOI:** 10.3892/or.2015.4086

**Published:** 2015-06-26

**Authors:** YUHUI HUANG, MOTOFUMI KUMAZOE, JAEHOON BAE, SHUHEI YAMADA, MIKA TAKAI, SHIORI HIDAKA, SHUYA YAMASHITA, YOONHEE KIM, YEONGSEON WON, MOTOKI MURATA, SHUNTARO TSUKAMOTO, HIROFUMI TACHIBANA

**Affiliations:** 1 Division of Applied Biological Chemistry, Department of Bioscience and Biotechnology, Faculty of Agriculture, Kyushu University, Higashi-ku, Fukuoka 812-8581, Japan; 2 Food Functional Design Research Center, Kyushu University, Higashi-ku, Fukuoka 812-8581, Japan

**Keywords:** green tea, epigallocatechin-*O*-gallate, chronic myeloid leukemia, lipid raft, acid sphingomyelinase

## Abstract

An epidemiological study showed that green tea consumption is associated with a reduced risk of hematopoietic malignancy. The major green tea polyphenol epigallocatechin-3-*O*-gallate (EGCG) is reported to have anticancer effects. Chronic myeloid leukemia (CML) is a major hematopoietic malignancy characterized by expansion of myeloid cells. In the present study, we showed EGCG-induced acid sphingomyelinase (ASM) activation and lipid raft clustering in CML cells. The ASM inhibitor desipramine significantly reduced EGCG-induced cell death. Protein kinase Cδ is a well-known kinase that plays an important role in ASM activation. We observed EGCG-induced phos-phorylation of protein kinase Cδ at Ser664. Importantly, EGCG-induced ASM activation was significantly reduced by pretreatment of CML cells with the soluble guanylate cyclase inhibitor NS2028, suggesting that EGCG induced ASM activation through the cyclic guanosine monophosphate (cGMP)-dependent pathway. Indeed, pharmacological inhibition of a cGMP-negative regulator enhanced the anti-CML effect of EGCG. These results indicate that EGCG-induced cell death via the cGMP/ASM pathway in CML cells.

## Introduction

Chronic myeloid leukemia (CML) is a major hematopoietic malignancy characterized by expansion of myeloid cells. In Western countries, CML accounts for 15–20% of all adult leukemias, and its prevalence in the US is predicted to increase from 70,000 in 2010 to 112,000 in 2020 to 181,000 cases in 2050 (1,2). The BCR-ABL tyrosine kinase provides the ideal molecular target for the therapy of CML (3,4). The use of imatinib or STI571, a tyrosine kinase inhibitor (TKI), drastically improved the prognosis of patients with CML. However, some portion of CML cells do not 'addict' to BCR-ABL and show very low sensitivity to TKIs widely used for CML treatment (5,6). These studies indicate the importance of developing a new therapeutic strategy for CML.

Green tea (*Camellia sinensis*) is widely consumed worldwide. Recent epidemiological studies have indicated a possible protective effect of green tea intake against the risk of hematopoietic malignancy (7–9). For example, based on a cohort study, in 41,761 Japanese adults aged 40–79 years, the risk of hematologic malignancy was negatively correlated with green tea consumption (9). The multivariate-adjusted hazard ratio of hematologic malignancies for five cups or more compared with <1 cup/day of green tea was 0.58 with a 95% confidence interval of 0.37–0.89 (9).

In a phase II clinical trial, green tea extract had an anticancer effect in patients with chronic lymphocytic leukemia (CLL) (10). Of 42 patients, 29 (69%) fulfilled the criteria for a biological response with a sustained ≥20% decline in ALC and/or a ≥30% reduction in the sum of the products of all nodal areas at some point during 6 months of treatment without severe adverse effects (10). Importantly, a green tea extract, polyphenon E, has been approved by the US Food and Drug Administration as the first botanical drug for the treatment of external genital and perianal warts (11).

Epigallocatechin-*O*-gallate (EGCG) is the predominant polyphenol catechin in green tea extract, and plays a central role in the anticancer effects of green tea polyphenols (12). Recent studies have shown that EGCG has anticancer effects in hematopoietic malignancy (13–16). However, several mechanisms have been suggested for EGCG-induced cell death (17–19) including the inhibition of anti-apoptosis protein, B-cell lymphoma (17), radical oxygen species (ROS) production (18) and VEGF receptor inhibition (19).

A previous model of plasma membranes assumed a homogeneous lipid bilayer randomly studded with membrane proteins (20). However, it has become clear that plasma membranes are heterogeneous and that clusters of lipids in a more ordered state are present within the generally disordered lipid environment of the membrane (20). These clusters are known as lipid rafts. Recent studies have shown that changes in membrane structure were induced in cancer cells treated with anticancer agents, including cisplatin (21). Notably, cisplatin increased in lipid raft cluster through acid sphingomyelinase (ASM) activation and the cluster increase plays a central role in its anticancer effect. In the present study, we show the impact of EGCG on lipid raft clustering. We also show that EGCG-induced ASM activation plays the crucial role in the anticancer effect of EGCG.

## Materials and methods

### Materials

Penicillin and streptomycin were purchased from Meiji Seika Pharma (Tokyo, Japan), fetal calf serum (FCS) was obtained from Biowest (Nuaillé, France). RPMI-1640 was obtained from Nissui Pharmaceutical Co. Ltd. (Tokyo, Japan). Catalase, EGCG, NS2028, superoxide dismutase (SOD), BODIPY-C12-sphingomyelin (SM) and desipramine were obtained from Sigma-Aldrich. Anti-PKCδ antibody was provided by Santa Cruz Biotechnology (Santa Cruz, CA, USA). Anti-phospho-PKCδ antibody at Ser664 antibodies was purchased from Abcam. Vardenafil was provided by TRC (Toronto, Canada). Bay 41-2272 was obtained from Enzo Life Sciences (Villeurbanne, France).

### Cell cultures and cell-based assay

The KU812 human CML cell line was provided by the Japanese Cancer Research Resources Bank (Tokyo, Japan) and maintained in RPMI-1640 supplemented with 10% (v/v) FCS, 100 U/ml penicillin and 100 *µ*g/ml streptomycin at 37°C in 5% CO_2_ at 100% humidity. To evaluate the anticancer effect of EGCG, KU812 cells were seeded in 24-well plates at 5×10^4^ cells/ml and treated with indicated concentrations of EGCG for 96 h in RPMI-1640 medium supplemented with 1% FCS, catalase (200 U/ml) and SOD (5 U/ml). A lipid-raft clustering assay was performed using two different fluorescent probe-tagged cholera toxin B subunits (Alexa Fluor^®^ 488 and Alexa Fluor^®^ 594 labeled cholera toxin B subunits) obtained from Life Technologies (Carlsbad, CA, USA). KU812 cells were stained with Alexa Fluor^®^ 488 and Alexa Fluor^®^ 594 labeled cholera toxin B subunits for 1 h on ice, and cells were treated at 37°C with EGCG for indicated times. All fluorescence images were captured with a fluorescence microscope (BZ-8100; Keyence). Measurement of ASM activity was performed as previously described (22). Briefly, KU812 cells were lysed in lysis buffer containing 50 mM Tris-HCl (pH 4.5), 150 mM NaCl, 1% Triton X-100, 1 mM EDTA, 50 mM NaF, 30 mM Na_4_P_2_O_7_, 1 mM phenylmethanesulfonyl fluoride, 2 mg/ml aprotinin and 1 mM pervanadate and incubated for 1 h at 4°C, followed by centrifugation at 15,000 × g for 15 min. The supernatant was incubated for 18 h at 37°C with substrate buffer (400 pmol BODIPY-C12-SM, 1% Triton X-100 and 200 mM sodium acetate in dH_2_O). The enzyme reaction was stopped by addition of chloroform:methanol [2:1 (v/v)].

### Western blot analysis

Cells were lysed in lysis buffer, and ~50 *µ*g of protein was suspended in Laemmli sample buffer (0.1 M Tris-HCl buffer, pH 6.8; 1% SDS; 0.05% mercaptoethanol; 10% glycerol; and 0.001% bromophenol blue), boiled and electrophoresed on SDS-polyacrylamide gels. Gels were electroblotted onto Trans-Blot nitrocellulose membranes (Bio-Rad) and incubated with the indicated antibodies in Tween-20 PBS (TPBS) containing 2.5% BSA. Blots were washed with TPBS and incubated in HRP-conjugated anti-rabbit or anti-mouse antibody.

### Statistical analyses

All data are presented as means ± SEM. Significance of differences between experimental variables was determined by Tukey's test. Statistical analyses were performed with KyPlot. A P-value of <0.05 was considered to indicate a statistically significant result. Isobologram analysis of growth inhibition was performed with CalcuSyn 2.0 software (Biosoft) as previously described (23).

## Results

### EGCG induces the reduction of viable cell numbers in the human CML cell accompanied with lipid raft clustering

**Figure 1:**
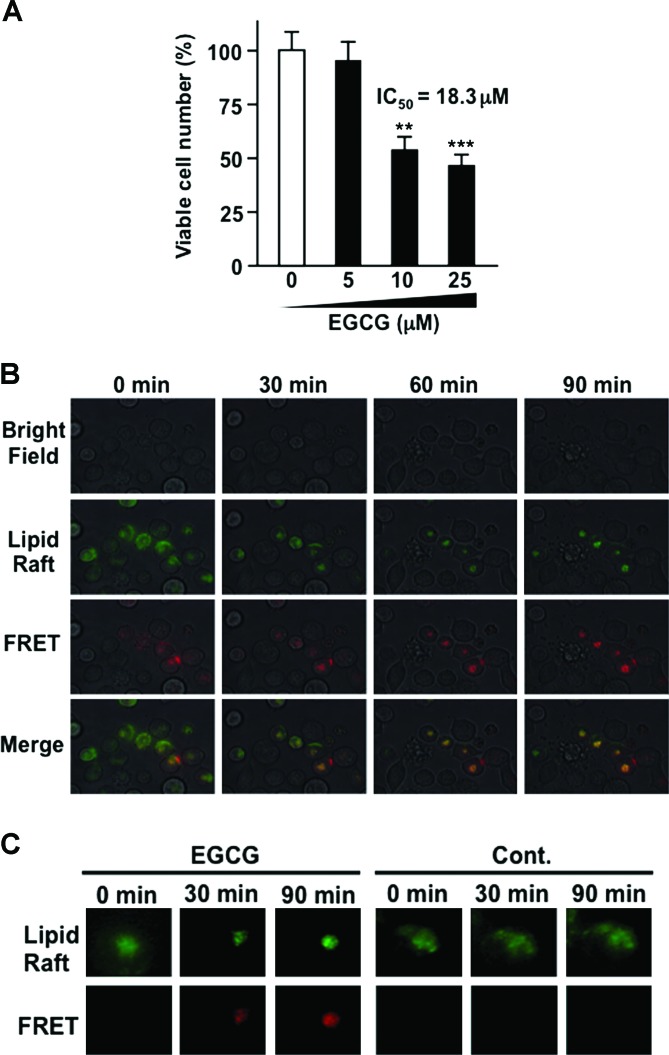
Epigallocatechin-*O*-gallate (EGCG) induces the reduction of viable cell number in human chronic myeloid leukemia (CML) cells, accompanied by lipid raft clustering. (A) KU812 cells were treated with EGCG for 96 h. The effect of EGCG on viable cell numbers was assessed by trypan blue staining. (B and C) The effect of EGCG on lipid rafts was observed by fluorescence microscopy. Data are means ± SEM (n=3). ^**^P<0.01, ^***^P<0.001.

We evaluated the anticancer effect of EGCG in the presence of SOD and catalase. Our results showed that EGCG treatment reduced the viable number of human CML KU812 cells in a dose-dependent manner. The 50% inhibitory concentration (IC_50_) of EGCG was ~18.3 *µ*M (Fig. 1A).

Since the resolution limit of fluorescence microscopy is ~200 nm, it is impossible to evaluate the size of lipid raft clusters by conventional method. Förster resonance energy transfer or fluorescence-detected resonance energy transfer (FRET), describing the energy transfer between two fluorescent probe molecules, has been applied as an important tool in structural biology (23). The efficiency of this energy transfer is inversely proportional to the sixth power of the distance between donor and acceptor, making FRET extremely sensitive to small changes in distance. The distance between the donor and the acceptor is typically in the range of 1–10 nm when FRET occurs. In the present study, we evaluated the lipid raft using both Alexa Fluor^®^ 488 and Alexa Fluor^®^ 594 labeled cholera toxin B subunits, well-known probes detecting ceramide-rich lipid rafts (21). In this system, when lipid rafts aggregate and form larger lipid rafts, FRET signaling is increased. Our results showed that in cells treated with 10 *µ*M of EGCG, the FRET signal increased in a time-dependent manner (Fig. 1B and C). In contrast, control cells showed no change (Fig. 1C). These results show that EGCG induced ceramide-rich lipid raft clustering in CML cells.

### EGCG, but not other EGCG-related compounds induces ASM activation in human CML cells

**Figure 2:**
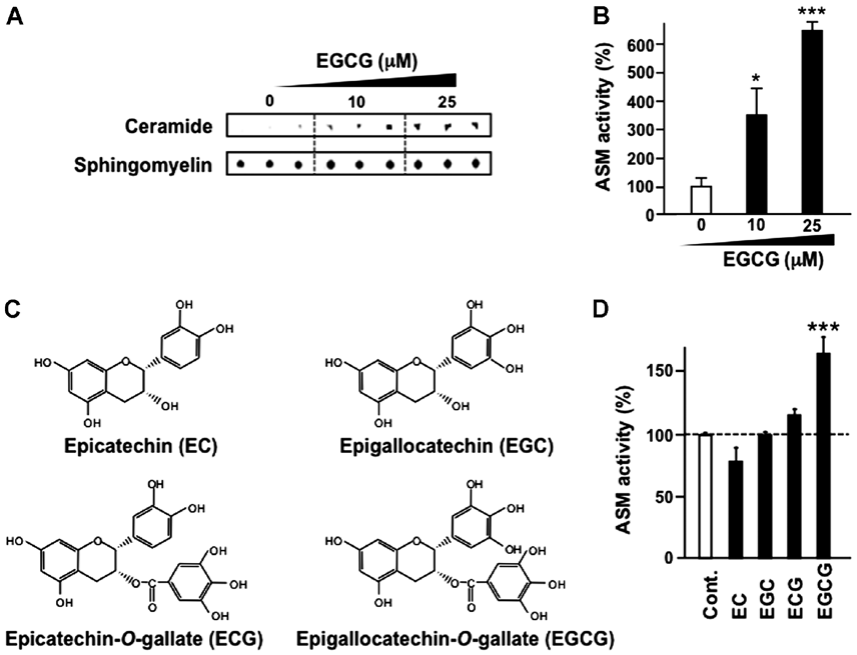
Epigallocatechin-*O*-gallate (EGCG), but not other EGCG-related compounds induces acid sphingomyelinase (ASM) activation in human chronic myeloid leukemia (CML) cells. (A and B) KU812 cells were treated with EGCG for 3 h and ASM enzyme activity was measured by TLC analysis. (C) Structures of EGCG and its analogs, epigallocatechin (EGC), epicatechin-*O*-gallate (ECG) and epicatechin (EC). (D) KU812 cells were treated with several catechins, including EGCG, EC, EGC and ECG for 3 h and ASM activity was evaluated. Data are means ± SEM (n=3). ^*^P<0.05, ^***^P<0.001.

KU812 cells were treated with EGCG for 3 h and ASM activity was evaluated by thin-layer chromatography. Our results suggested that EGCG treatment increased ASM activity in a dose-dependent manner (Fig. 2A and B).

The major green tea catechins are EGCG, epicatechin (EC), epigallocatechin (EGC) and epicatechin-3-gallate (ECG) as shown in Fig. 2C. These major tea catechins are characterized by dihydroxyl or trihydroxyl substitutions on the B ring and the *m*-5,7-dihydroxyl substitutions on the A ring. The B ring appears to be the principal site of antioxidant reactions, and the antioxidant activity is further increased by the trihydroxyl structure on the D ring (gallate) in EGCG and ECG (24). Notably, other tea catechins, including EC, EGC and ECG, structurally related compounds, did not affect ASM activity, whereas EGCG activated ASM in KU812 cells (Fig. 2D).

### ASM plays the crucial role in the anti-CML effect of EGCG

**Figure 3:**
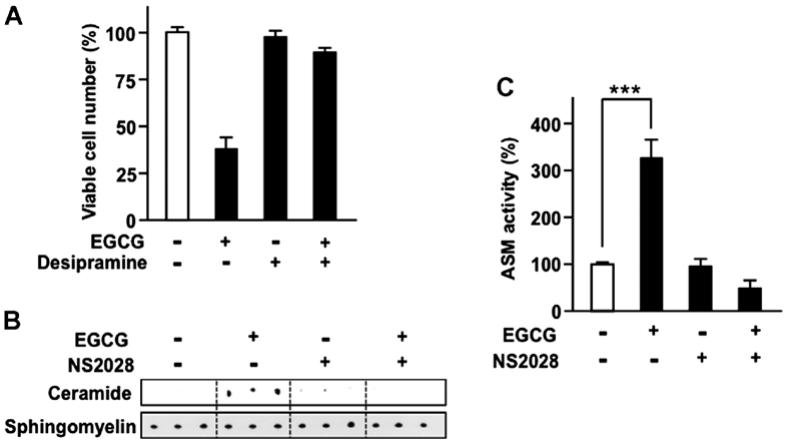
Acid sphingomyelinase (ASM) plays a crucial role in antichronic myeloid leukemia (CML) effect of epigallocatechin-3-*O*-gallate (EGCG). (A) KU812 cells were pretreated with or without ASM inhibitor, desipramine 5 *µ*M for 3 h and cells were treated with EGCG (10 *µ*M) for 96 h. Viable cell numbers were measured by trypan blue staining. Data are means ± SEM (n=3). (B and C) KU812 cells were cultured with or without the soluble guanylyl cyclase inhibitor NS2028 (5 *µ*M) for 3 h. KU812 cells were treated with EGCG (10 *µ*M) for 3 h and ASM activity was evaluated by TLC analysis. Data are means ± SEM (n=3). ^**^P<0.01, ^***^P<0.001.

We found that EGCG induced the reduction of viable cell number in CML accompanied with lipid raft clustering (Fig. 1A and B). ASM activation is a well-known mechanism in cisplatin-induced lipid raft clustering (21,25). We also found that EGCG, yet not other structurally related compounds, activated ASM (Fig. 2D). To determine the role of ASM in EGCG-induced viable cell reduction, we evaluated the effect of the ASM inhibitor desipramine, a tricyclic antidepressant on the anti-CML effect of EGCG (Fig. 3A). Our results showed that desipramine significantly reduced anti-CML effect of EGCG, suggesting that ASM plays the central role in the anti-CML effect of EGCG.

The 67-kDa laminin receptor (67LR) is the molecular target of EGCG (26). We previously reported that EGCG induced cell death in multiple myeloma cells through activation of the cell surficial protein 67-LR (14). Several studies have shown that 67LR also mediates the effects of EGCG, including an anti-acute myeloid leukemia (13), an anticervical cancer (27) and antimelanoma effects (28,29).

Cyclic guanosine monophosphate (cGMP) is one of the secondary messengers that plays the central role in the regulation of vascular homeostasis and sexual arousal-induced penile erection. Regulation of cGMP is a well-established strategy for vasodilation and increased blood flow. Soluble guanylyl cyclase (sGC) is an enzyme involved in EGCG-induced cGMP upregulation (14). We previously reported the role of cGMP as the secondary messenger that transmits EGCG-induced 67LR-dependent apoptosis (14). Indeed, EC, EGC and ECG that have little affinity for 67LR (26) did not affect the intracellular cGMP level (14). Since EC, EGC and ECG, which have low affinity to 67LR, did not affect ASM activity, whereas EGCG activated ASM in KU812 cells (Fig. 2D), we hypothesized that EGCG increased ASM activity via an sGC-dependent mechanism. To assess the role of sGC in EGCG-induced ASM activation, we pretreated CML cells with the sGC inhibitor NS2028 before treatment with EGCG (Fig. 3B and C). NS2028 pretreatment significantly reduced EGCG-induced ASM activation. These findings suggested that EGCG-induced ASM activation via sGC-dependent mechanisms.

### EGCG activates PKCδ attributed to ASM activation in CML

**Figure 4:**
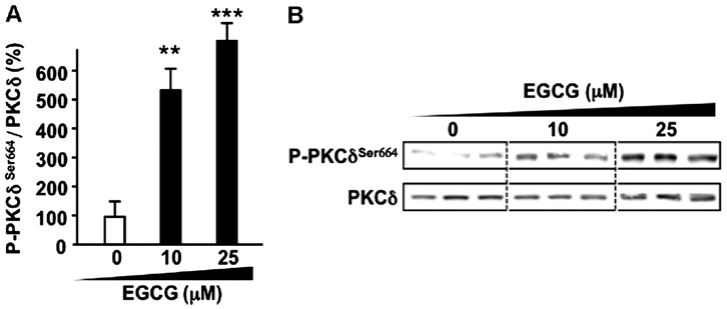
Epigallocatechin-*O*-gallate (EGCG) activates PKCδ attributed to ASM activation in chronic myeloid leukemia (CML). (A and B) KU812 cells were treated with EGCG for 3 h and phosphorylation of PKCδ at Ser664 was evaluated by western blot analysis. Data are means ± SEM (n=3). ^**^P<0.01, ^***^P<0.001.

Previous studies suggested that PKCδ is one of the novel PKCs activated by diacylglycerol or 12-otetradecanoylphorbol 13-acetate. PKCδ is well known as a kinase that acts as a trigger of ASM activation. In fact, the PKCδ knockout mouse shows a phenotype of dysfunction in UV-induced ASM-dependent apoptosis (30). To assess the effect of EGCG on the phos-phorylation of PKCδ at Ser664 that involved apoptotic cell death (31) was measured. EGCG induced phosphorylation of PKCδ at Ser664 in a dose-dependent manner (Fig. 4A and B).

## Discussion

A previous study showed that EGCG killed hematopoietic malignancy cells by the production of ROS in an *in vitro* model (18). Although the vicinal trihydroxy structure of EGCG contributes to these antioxidative activities of tea polyphenols, it also renders these compounds susceptible to air oxidation at alkaline or even neutral pH (24,25). Autooxidation leads to the generation of superoxide anions and H_2_O_2_ (12). Several studies have also shown that high concentrations of EGCG induce ROS-dependent cell death (12,32). The oxygen partial pressure in the internal organs is normally much lower than that under cell culture conditions (<40 vs. 160 mm Hg), and cells contain antioxidative enzymes such as SOD and glutathione peroxidase (12), recent studies recommend the use of SOD and catalase to halt EGCG-induced ROS production to avoid artifacts (12,32). In the presence of SOD and catalase, EGCG also induced significant anticancer activities (14,16,22,27–29,33), suggesting that the effects of EGCG on cancer cells are independent of ROS. Our data showed that by co-treatment with SOD and catalase, EGCG exerted its anti-CML effect in a sGC-dependent ASM activation pathway.

A lipid raft consists of mostly saturated hydrocarbon chains with several kinds of tightly intercalated sphingolipids and cholesterol organized the liquid-ordered state in plasma membranes (34). Lipid rafts play an essential role in the regulation of various signaling, cell growth, and apoptosis. Proteins located in lipid rafts include glycosylphosphatidylinositol–anchored proteins, doubly acylated proteins such as Src-family kinases or α-subunits of heterotrimeric G proteins, cholesterol-linked and palmitoylated proteins such as Hedgehog, epidermal growth factor receptor (EGFR) and transmembrane proteins, particularly palmitoylated ones (34). Several studies indicate that EGCG affects lipid raft function (35,36) in its anticancer effect. Adachi *et al* reported that EGCG has an inhibitory effect on activation of EGFR via reduction of the lipid (35). In that study, EGCG reduced cholesterol-rich lipid rafts in a dose-dependent manner (35). As a result, EGCG drastically reduced epidermal growth factor-induced EGFR phosphorylation, which plays the crucial role in tumor cell growth and survival. However, little is known about the effect of EGCG on lipid raft clustering in CML cells. In the present study, we showed that EGCG induces lipid raft clustering in CML.

Ceramide and its metabolites influence cellular processes that include apoptosis, autophagy and inflammation (37). Enzymes of sphingolipid metabolism determine cellular levels of ceramide, so that knowledge of the regulation of these enzymes provides insight into the cellular mechanisms underlying ceramide generation, accumulation and action. Ceramide can be generated by hydrolysis of complex sphingolipids or by the recently characterized ceramide salvage pathway. ASM, also known as sphingomyelin phosphodiesterase 1 (SMPD1), is a member of the SMPD family and occupies a prominent position in sphingolipid catabolism, catalyzing the hydrolysis of sphingomyelin to ceramide and phosphorylcholine. In a recent study, ASM-null mice were protected against a variety of stress stimuli, including Fas ligand, lipopolysaccharide, ionizing radiation and photocytotoxicity, ischemia/reperfusion injury, cisplatin and tumor necrosis factor-α, as a result of impaired ceramide generation. Notably, previous studies (21,25) showed that cisplatin, the first member of a class of platinum-containing anticancer drugs, induced apoptosis through ASM activation and thereby caused ceramide-dependent lipid raft clustering. These findings initiated our interest to investigate the effect of EGCG on ASM activity. Importantly, ASM activation was induced by EGCG, whereas its analog could not induce ASM activation, showing that this pathway is specifically activated by EGCG.

67LR is highly upregulated in hematopoietic malignancies, including multiple myeloma (14), acute myeloid leukemia (13) and CLL (16), compared with normal peripheral blood mono-nuclear cells (PBMCs). Indeed, EGCG selectively kills those cancer cells without affecting normal PBMCs (13,14,33). Thus, EGCG selectively suppresses CML cells without affecting normal cells. In the last 3 years, severe adverse effects of the second- and third-generation TKIs have been reported (1). These findings suggest EGCG as a choice for the CML treatment.

Furthermore, we have reported that cGMP transmits an anticancer effect and that the presence of a negative regulator of cGMP protects against EGCG-induced cell death (14,23). Indeed, the present study based on multiple myeloma cells showed that cGMP production is the 'choke point' of the anticancer effect of EGCG (14). Moreover, we reported that phosphodiesterase 5 (PDE5) inhibition synergistically enhanced the anticancer effect of EGCG in multiple myeloma (14) and acute myeloid leukemia cells (33). These data suggested that pharmacological inhibition of a sGC negative regulator could be an ideal approach to enhance the anti-CML effect of EGCG.

In conclusion, the present study demonstrated that EGCG-induced lipid raft clustering in human CML cells. Indeed, the present study further reveals that EGCG induced the cell death via the sGC/ASM pathway. The present study clarifies the molecular mechanism of EGCG in CML, and suggests that EGCG as a choice for the CML treatment and pharmacological inhibition of a sGC negative regulator could be an ideal approach to enhance the anti-CML effect of EGCG.
